# Stimulating neural plasticity with real‐time fMRI neurofeedback in Huntington's disease: A proof of concept study

**DOI:** 10.1002/hbm.23921

**Published:** 2017-12-13

**Authors:** Marina Papoutsi, Nikolaus Weiskopf, Douglas Langbehn, Ralf Reilmann, Geraint Rees, Sarah J Tabrizi

**Affiliations:** ^1^ UCL Huntington's Disease Centre, Institute of Neurology, University College London London United Kingdom; ^2^ Max Planck Institute for Human Cognitive and Brain Sciences Leipzig Germany; ^3^ Wellcome Trust Centre for Neuroimaging Institute of Neurology, University College London London United Kingdom; ^4^ Carver College of Medicine University of Iowa Iowa City Iowa; ^5^ George Huntington Institute and Department of Radiology University of Muenster Münster Germany; ^6^ Section for Neurodegeneration and Hertie Institute for Clinical Brain Research, University of Tuebingen Tübingen Germany; ^7^ Institute of Cognitive Neuroscience, University College London London United Kingdom

**Keywords:** brain training, Huntington's disease, neurodegenerative diseases, neurofeedback training, neuroplasticity, real‐time fMRI

## Abstract

Novel methods that stimulate neuroplasticity are increasingly being studied to treat neurological and psychiatric conditions. We sought to determine whether real‐time fMRI neurofeedback training is feasible in Huntington's disease (HD), and assess any factors that contribute to its effectiveness. In this proof‐of‐concept study, we used this technique to train 10 patients with HD to volitionally regulate the activity of their supplementary motor area (SMA). We collected detailed behavioral and neuroimaging data before and after training to examine changes of brain function and structure, and cognitive and motor performance. We found that patients overall learned to increase activity of the target region during training with variable effects on cognitive and motor behavior. Improved cognitive and motor performance after training predicted increases in pre‐SMA grey matter volume, fMRI activity in the left putamen, and increased SMA–left putamen functional connectivity. Although we did not directly target the putamen and corticostriatal connectivity during neurofeedback training, our results suggest that training the SMA can lead to regulation of associated networks with beneficial effects in behavior. We conclude that neurofeedback training can induce plasticity in patients with Huntington's disease despite the presence of neurodegeneration, and the effects of training a single region may engage other regions and circuits implicated in disease pathology.

## 1 INTRODUCTION

Neurological disease symptoms are associated with neuronal dysfunction, that is, atrophy, increased (Filippini et al., 2009) or decreased activity, or connectivity (Burciu et al., 2016). Novel methods that induce neuroplasticity could normalize neuronal function and may improve symptoms or slow disease progression. Real‐time fMRI neurofeedback training is a novel approach that uses real‐time fMRI and reinforcement learning to induce changes in brain activity (Caria, Sitaram, & Birbaumer, 2012; De Charms, 2008; Linden and Turner, 2016; MacInnes, Dickerson, Chen, & Adcock, 2016; Shibata, Watanabe, Sasaki, & Kawato, 2011; Sulzer et al., 2013; Weiskopf, 2012). It improves cognitive function in healthy individuals (De Bettencourt, Cohen, Lee, Norman, & Turk‐Browne, 2015; Scharnowski, Hutton, Josephs, Weiskopf, & Rees, 2012) and motor function in patients with Parkinson's disease (Subramanian et al., 2011, 2016). By providing participants with feedback of their own neural activity in a closed‐loop experimental design, participants gradually learn to control it, thereby inducing structural and functional changes to target regions and their associated networks (Greer, Trujillo, Glover, & Knutson, 2014; Haller et al., 2013; Horovitz, Berman, & Hallett, 2010; MacInnes et al., 2016; Megumi, Yamashita, Kawato, & Imamizu, 2015; Ruiz et al., 2013).

**Figure 1:**
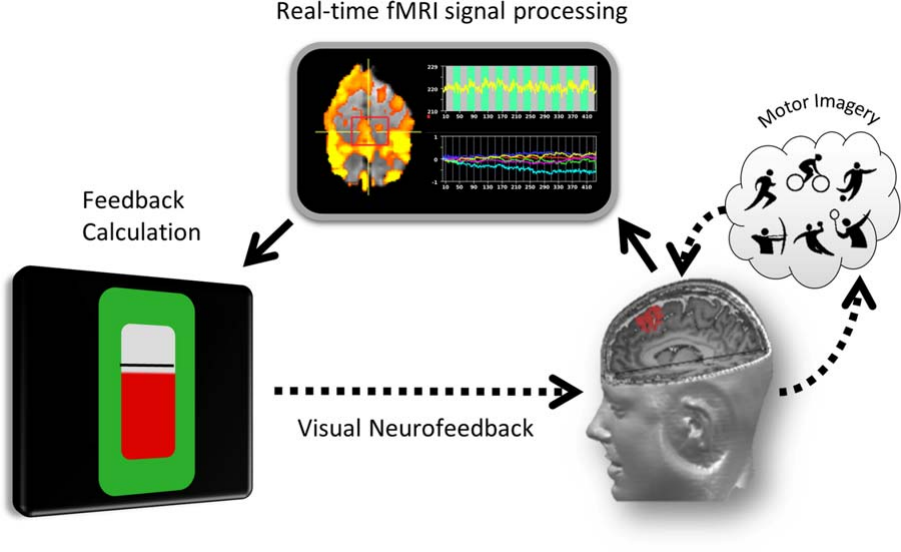
Schematics of the neurofeedback training setup. Continuous feedback in the form of a red bar representing the BOLD signal recorded from the target ROI (here, SMA) is rear‐projected onto a screen viewed by the participant while they are lying in the MRI scanner. SMA signals are recorded using the MRI scanner, analyzed in near real‐time using Turbo‐BrainVoyager and converted into a visual representation using custom MATLAB scripts. The height of the red bar reflects the magnitude of the BOLD signal from the SMA during upregulation compared to baseline. The participants are instructed to attempt to use motor imagery (or any other strategy that works) to increase the height of the bar as much as they can. The frame around the bar turns green and the black line goes up to signal to the participants to upregulate and increase the height of the red bar. SMA = supplementary motor area

For Huntington's disease (HD), an autosomal‐dominant neurodegenerative disease affecting motor and cognitive function, regions affected by disease pathology typically show atrophy and reduction of activity and connectivity that correlates with impairment (McColgan et al., 2015; Novak et al., 2012, 2015; Poudel et al., 2014; Tabrizi et al., 2009, 2011; Wolf, Vasic, Schönfeldt‐Lecuona, Landwehrmeyer, & Ecker, 2007; Wolf et al., 2012). An important question is therefore whether HD patients can learn to volitionally regulate the activity of affected regions, and what effect that would have on their brain activity after training, and their cognitive and motor performance. To answer this question, we recruited ten HD patients to take part in an intensive real‐time fMRI neurofeedback training study that consisted of at least 3 neurofeedback training visits on separate days. Patients were trained to volitionally increase BOLD fMRI signals from the Supplementary Motor Area (SMA) by receiving near real‐time visual feedback in the form of a thermometer bar whose height represented the BOLD signal recorded from SMA (Figure 1). We also examined changes in their brain function and structure, as well as their cognitive and motor performance before and after training. The aim of this proof‐of‐concept study was to determine whether neurofeedback training is feasible in HD, and to identify any parameters that were associated with improvement of patient performance in order to inform future randomized controlled trials.

As part of the training, patients used motor imagery and received continuous feedback reflecting the level of BOLD activity of the SMA (including pre‐SMA). We selected the SMA, because its function and connectivity with the striatum is affected by HD (Klöppel et al., 2009) and can provide reliable, continuous signal estimates suitable for real‐time fMRI neurofeedback training (Subramanian et al., 2011, 2016). We expected that patients' SMA activity would increase during the training and after the training patients would be able to volitionally regulate their SMA activity, even in the absence of neurofeedback (Haller et al., 2013; Scharnowski et al., 2012). To the extent that learnt control of SMA activity is beneficial, we expect that it would have a positive effect on the patients' behavioral performance. Changes in structural MRI measurements have not been previously reported following neurofeedback training; however, other types of training paradigms have reported changes in grey and white matter (Draganski et al., 2004; Lewis, Baldassarre, Committeri, Romani, & Corbetta, 2009; Sagi et al., 2012; Sampaio‐Baptista et al., 2013, 2014; Takeuchi et al., 2010) following training and we were therefore interested in examining whether such effects would be present in regions modulated during training.

Previous studies using neurofeedback training have shown that the changes induced by neurofeedback are not just localized to the training target region, but extend to other areas connected to the target region or engaged by the neurofeedback training task (Emmert et al., 2016; Haller et al., 2013; Horovitz et al., 2010; Koush et al., 2013; Ruiz et al., 2013; Zhang, Yao, Shen, Yang, & Zhao, 2015; Zotev et al., 2011). Using real‐time fMRI based neurofeedback instead of EEG‐based neurofeedback enables us to monitor changes in functional brain activity that occur during training, not only in the target region, but the whole‐brain, including sub‐cortical regions such as the striatum, which is commonly recruited during neurofeedback training (Emmert et al., 2016). Corticostriatal connectivity is disrupted in HD and associated with cognitive and motor impairment (Bohanna, Georgiou‐Karistianis, & Egan, 2011; McColgan et al., 2015; Novak et al., 2015; Tabrizi et al., 2009, 2011), we were therefore interested to examine whether learning to regulate SMA activity would lead to changes in striatal function and connectivity with the SMA.

The effect of interventions such as multidomain cognitive training extend to untrained tasks or everyday aspects (Anguera et al., 2013; Subramaniam et al., 2012; Willis et al., 2006), an effect known as “transfer,” whereby improvements on cognitive domains relevant to a disease can be generalized to other domains of cognition and general quality of life. The exact mechanism of transfer is, however, not well understood and could be related to both targeting the pathology and stimulating neuroplasticity (Mishra and Gazzaley, 2014). Neurofeedback training is primarily based on reinforcement learning, but also includes visual processing and decision making (Haller et al., 2013; Lawrence et al., 2014). Here, by using the SMA as the target region, motor control networks were also engaged. It is therefore possible, that neurofeedback training could have a beneficial effect on untrained cognitive and motor tasks, especially if those tasks are associated with brain regions and networks that are engaged during the training. Although the aim of this study was not to prove efficacy, but feasibility, preliminary evidence was collected on cognitive and motor tasks before and after training using untrained, independently validated biomarkers of HD progression (Tabrizi et al., 2009, 2012, 2011, 2013). This allowed us to gain insight on whether such methods hold promise as an intervention in HD, and to identify the factors associated with any improvement in patient performance.

In summary, the aim of this study was to examine whether HD patients are able to learn to regulate their SMA activity using real‐time fMRI neurofeedback training and what effect that would have on their motor and cognitive performance. In addition, we were interested to understand which changes in brain function would correlate with improvement in behavior, which is important to improve the design of future trials.

## 2 MATERIALS AND METHODS

### 2.1 Participants

Eleven adults were recruited to the study. One participant withdrew from the study after two visits for reasons unrelated to the study; the data were not used in any of the analyses. The remaining 10 participants completed the training and testing protocol (7 females, 3 males; mean (SD) age = 51.1 (9.4); see Table 1 for detailed participant information). All participants reported that they were right‐handed and all had undergone genetic testing for HD with a positive result. Because large head movements cause severe artefacts in the MRI signal, participants were selected with none‐to‐mild motor signs. To take part in the study, they had to have a Unified Huntington's Disease Rating Scale (Huntington Study Group, 1996) (UHDRS) Total Motor Score (TMS) of <20 and a chorea subscore of <5. The UHDRS assessment was performed by trained clinicians from the HD Multidisciplinary Clinic at the National Hospital for Neurology and Neurosurgery (NHNN) <12 months before recruitment to the study. None of the participants recruited were prescribed dopamine receptor antagonists, for example, tetrabenazine or olanzapine, which could disrupt reinforcement learning. At the first visit participants also completed the Montreal Cognitive Assessment test (Nasreddine et al., 2005) (MOCA), which gave an estimate of the participants' level of cognitive capacity. All participants provided written informed consent according to the Declaration of Helsinki and the study was approved by the local Research Ethics Committee.

**Table 1:** Participant characteristics

Participant	Gender	Age	CAG repeat length	CAG age product (CAP)	Caudate volume (%ICV)	Montreal Cognitive Assessment (MOCA)	UHDRS		
							*Total Motor Score (TMS)*	*Diagnostic ConfidenceScore (DCS)*	*Total Functional Capacity (TFC)*
**1**	F	43	46	110	0.29	22	14	4	13
**2**	F	56	40	89	0.53	30	4	3	12
**3**	F	46	44	104	0.39	26	16	4	11
**4**	F	68	39	98	0.45	27	11	4	12
**5**	M	48	45	115	0.33	29	13	4	12
**6**	F	59	42	112	0.36	28	12	4	13
**7**	F	61	40	97	0.48	27	5	1	13
**8**	F	43	41	76	0.47	28	0	0	13
**9**	M	48	44	108	0.41	23	17	4	12
**10**	M	39	43	81	0.46	29	8	3	13

*Note*. Abbreviations: ICV = intracranial volume; UHDRS = Unified Huntington's Disease Rating Score. Normalized CAG Age Product (CAP score) was calculated as 100 × Age × (CAG − 30)/627, based on the formula by Ross et al. (2014), such that the CAP score is ∼100 at an HD gene‐carrier's expected age of onset as estimated by Langbehn et al. (2004).

To identify and exclude data with large motion related artefacts or other issues, thorough checks were performed. Data that did not fit the criteria specified in detail in the Methods section below were excluded. From some of the analyses, we excluded participants; however, the main analyses included in this article include all 10 patients. In more detail, all 10 patients were used in the analyses testing for training effects (i.e., the change in the target ROI activity from the first to the last training session). All 10 patients were also used in the analyses looking at change in cognitive and motor performance before and after training (composite score). All 10 patients were included in the regression analyses with performance (i.e., composite score) and change in BOLD signal from the first to the last training session, and the regression analyses between the composite score and change in connectivity between the target ROI and left putamen and the rest of the brain. One patient was excluded from the VBM and VBQ analyses because of head motion, that is, nine patients were used in this analysis. One patient was excluded from the fMRI paced tapping task analyses, because of poor performance during the tapping task (Supporting Information), that is, nine patients were used in this analysis. One additional patient was excluded from the analyses of the behavioral data of the fMRI tapping task and the regression analyses between the BOLD signal and the tapping task performance, because the data collected from the button box were not reliable enough to perform the required analyses. Therefore only eight patients were used in these analyses.

### 2.2 Study protocol

As part of the study, participants completed 1–2 pretraining, 3–4 neurofeedback training, and 1–2 post‐training visits, depending on the participants' availability and preference. To take part in the study, participants had to commit to complete at least three training visits (the fourth visit was optional) in addition to the baseline and follow‐up visits. Each visit was 3 days to 2 weeks apart. Two participants completed only three training visits, whereas the remaining eight were able to complete four. The mean (±SD) period between consecutive visits was 8.8 (±7.9) days and the mean (±SD) period from the first to the last visit was 53.7 (±17.4) days. A diagram of the study design is shown in Supporting Information, diagram 1.

#### 2.2.1 Neurofeedback training visits

The neurofeedback training visits started with an fMRI fist clenching task with the nondominant hand, which consisted of 21 blocks of rest followed by fist clenching with 20 s duration per block. This was used as a functional localizer for the supplementary motor area (SMA) bilaterally (Subramanian et al., 2011) and was analyzed in real‐time using Turbo‐BrainVoyager (TBV; Brain Innovation, The Netherlands). A region of interest (ROI) was drawn for the contrast fist clenching versus rest selecting the voxels with activation greater than a *t* value of 3 in the SMA (including pre‐SMA) bilaterally (Figure 1 and Supporting Information, Figure 1). The selected ROI was then used as target for the subsequent neurofeedback training runs. The ROI was redrawn at every visit for each participant, which ensured that only the highest activated voxels in the SMA were used for neurofeedback at each training visit and for each participant. This is comparable to the approach used in other studies, where the neurofeedback signal presented in each block is calculated from the highest activated voxels within the same region (Nicholson et al., 2017; Paret et al., 2014, 2016; Subramanian et al., 2011) or across the whole brain (Linden et al., 2012), and thereby the voxels included in the calculation of the neurofeedback signal are different in every trial, but most relevant for neurofeedback training.

The participants performed 4 neurofeedback training runs per visit (one participant on one of the training visits only completed 3 training runs, because of a technical failure). Prior to scanning, the participants were instructed to refrain from making any overt movements and use motor imagery, or any other mental strategy they felt is effective, to increase the activation of the target ROI during the upregulation blocks. To inspect the participants' limb movements during the functional localizer and neurofeedback training runs and ensure task compliance, pneumatic tubes connected to a pressure sensor were taped along the participants' palms and feet. This setup enabled the detection of gross limb movements, for example, flexing one's fingers or squeezing one's fist, during the scanning session and ensured task compliance (see paragraph on data quality control on the Supporting Information).

The neurofeedback training runs consisted of 6 baseline blocks, 6 response blocks, and 5 upregulation blocks, presented in interleaved order. The baseline and upregulation blocks were 30 s long, the response blocks were 16 s to allow for the hemodynamic response to return to baseline prior to the upregulation blocks. To prevent participants from engaging in motor imagery during the baseline blocks and to provide an appropriate control condition, the baseline blocks consisted of a simple visual attention task that was closely matched to the neurofeedback training blocks. During the baseline blocks, the participants monitored changes in the luminance of the white bar, if the white bar flickered to grey (for 1 s), they would wait until a question mark appeared inside the white bar and then make a response by clenching their left (nondominant) fist once (Supporting Information, Figure 2). A maximum 3 out of 6 baseline blocks flickered per run and the timing of the flickering during the block was random.

During the upregulation blocks, neurofeedback reflecting the level of target ROI activation was presented visually as a red thermometer bar (Subramanian et al., 2011) (Figure 1 and Supporting Information, Figure 2). The height of the thermometer bar updated on average every 1.5 s and indicated the target ROI percent signal change (PSC) at any given point during upregulation compared to the mean activity of the preceding baseline block. A black line was set at 3/5 of the thermometer height and acted as an additional reminder to the participant that they needed to increase the height of the red bar. To calculate the baseline, we averaged the target ROI activity during the preceding baseline block weighted by a mixture of two gamma functions modelling the hemodynamic response during the 30 s baseline block. The activity during the response blocks was not included in the estimation of the baseline activity.

To facilitate learning and control participant levels of anxiety and motivation, we introduced shaping, whereby the difficulty in increasing the height of the thermometer bar was adjusted in response to the participants' performance (Linden et al., 2012; Weiskopf et al., 2004). In more detail, the maximum height of the thermometer bar was scaled by the maximum PSC achieved during the preceding upregulation block. If participants' PSC during an upregulation block was low, the maximum height of the bar for the next block would be set low. Therefore, small increases in PSC would appear larger during the next block, rewarding positive results, even if low. On the other hand, if participants achieved a large increase in the target ROI PSC, they would need to achieve a higher PSC in the next block to be rewarded with a higher than before increase in the height of the thermometer bar. Only PSC increases were fed back to participants, decreases were not shown. In this way, participants were positively reinforced to continuously improve by adjusting the level of difficulty in each block, but without becoming demoralized, even if they underperformed during a block.

#### 2.2.2 Pre‐ and post‐training visits

The pre‐ and post‐training sessions included (a) a selection of cognitive and quantitative motor (Q‐Motor) assessments sensitive to HD progression from the Track‐HD assessment battery (Tabrizi et al., 2009, 2012, 2011, 2013) (see paragraph below on measures of cognitive and motor performance for details of the tasks included), (b) Multi‐Parameter Maps to assess structural MRI plasticity (Weiskopf et al., 2013), and (c) an fMRI paced finger‐tapping task with the left (nondominant) index finger. To minimize practice effects when comparing pre‐ and post‐training performance, the cognitive and Q‐Motor assessments were repeated prior to the first neurofeedback training session. The second assessment was the baseline visit and compared against the post‐training performance.

The paced tapping task that was performed during the pre‐ and post‐training visits consisted of alternating blocks of rest and paced tapping with the left (nondominant) index finger at 1.8 Hz (11 blocks per run with 20 s duration per block). The pacing tone was on during both the rest and tapping blocks, but participants were instructed to tap only during the tapping blocks, that is, when the frame was green (Supporting Information, Figure 2).

The post‐training visits included two versions of the paced tapping task, with and without upregulation. The two types of runs where identical in their presentation and participants were informed by the experimenter before the start of the run whether they should upregulate or not during the upcoming run. During the runs with upregulation, the participants were asked to upregulate activity within their target ROI while tapping, but without receiving any neurofeedback. The instructions provided regarding upregulation, were to apply the strategy they felt was the most successful in getting the bar high during the neurofeedback training runs. The paced tapping runs with upregulation were designed to test whether participants were able to volitionally upregulate activity within the target ROI in the absence of neurofeedback and the effect that this will have on their performance (Scharnowski et al., 2012). The presentation order of the runs was counterbalanced across participants and visits with half of the sessions starting with the tapping with upregulation runs.

#### 2.2.3 fMRI task setup

Stimulus presentation and response recording was controlled using in‐house MATLAB (Mathworks) scripts and the Cogent toolbox (http://www.vislab.ucl.ac.uk/cogent_2000.php). Turbo‐BrainVoyager (Brain Innovation, The Netherlands) was used to record the target ROI activity during the neurofeedback training visits. Spike2 (CED) was used to record participant limb movements using the pneumatic tubes, and breathing and heart rate. Etymotic earphones (Etymotic ER‐3A) were used to deliver auditory stimuli.

Stimulus presentation was consistent across all fMRI tasks—paced tapping, fist clenching and neurofeedback training. The “active” blocks, that is, the paced tapping, fist clenching and upregulation blocks, consisted of a white bar inside a green frame, whereas the “baseline” blocks consisted of a white bar inside a grey frame (Supporting Information, Figure 2). Our rationale was to condition participants accordingly to increase motor activation only during the “active” blocks (green frame), thereby ensuring greater task compliance. This was important for the neurofeedback training runs, where the participants were instructed to increase brain activity only during the upregulation blocks and rest during the baseline blocks. All fMRI tasks were practiced prior to each scanning session. More details on the exact setup of the tasks for each of the sessions are presented in the Supporting Information, methods.

### 2.3 Measures of cognitive and motor performance (composite score)

Selected cognitive and Q‐Motor measures, independently validated as sensitive to disease progression in HD (Tabrizi et al., 2009, 2012, 2011, 2013), were used to assess changes in cognitive and motor performance following neurofeedback training. Because we did not have an a priori hypothesis about which of these measures would be affected by the training, but were instead interested in identifying changes in overall cognitive and motor capacity, we combined all the selected measures into one composite score. The measures collected were objective measurements of cognitive and motor performance and suitable for patients at both pre‐ and early symptomatic stages of HD.

In more detail, the cognitive measurements included were number correct for Stroop Word Reading only, number correct for Symbol Digit Modalities Test (SDMT), annulus length for Indirect Circle Tracing (log transformed), and number correct for negative Emotion Recognition. The Q‐Motor measurements included were inter‐tap interval (ITI) and standard deviation of inter‐onset interval (log transformed; log SD IOI) during speeded tapping with the left (nondominant) index finger, and standard deviation of mid‐tap interval deviation from target rhythm (log transformed; log SD dMTI) for paced tapping with left index finger at 1.8 Hz. To harmonize the direction of change across all measures, so that a larger number equates better performance, ITI, log SD IOI, and log SD dMTI were converted to negative. The selected measures were then standardized using the mean and standard deviation from an independent sample of early HD patients (Track‐HD visit 2 (Tabrizi et al., 2011)). The mean of each participant's standardized values was then used as the composite score of Q‐Motor and cognitive performance for all visits.

The composite score at the baseline visit correlated highly with the normalized CAG Age Product score (CAP score; Spearman's *r* = −.80, *p* = .010), the MOCA (Spearman's *r* = .78, *p* = .014), and UHDRS TMS (Spearman's *r* = −.88 and *p* = .002) after controlling for age (all results were also significant without controlling for age, all *p* < .02). It was therefore a sensitive measure of the participant's disease stage and overall cognitive and motor capacity.

### 2.4 Measures of disease pathology

Caudate volume (as percent of intracranial volume; ICV) was used as an indirect measure of disease pathology (Klöppel et al., 2015). Caudate atrophy is a characteristic sign of disease onset, which can be detected many years before symptom onset and is very sensitive to disease progression (Tabrizi et al., 2009, 2012, 2011, 2013). It is therefore an appropriate measure of disease pathology to be used in both pre‐ and manifest HD patients. Our measure of caudate volume correlated highly with both the CAP score (Ross et al., 2014), the UHDRS TMS, as well as the composite score at the baseline visit (Spearman's *r* = −.86, *r* = −.77 and *r* = .81 respectively, all *p* < .02).

### 2.5 MRI acquisition parameters

For all the fMRI tasks, we used a whole‐brain multi‐shot 3D echo‐planar imaging (EPI) sequence (Lutti, Thomas, Hutton, & Weiskopf, 2013) with TR = 1 s, TE = 29.5 ms, excitation flip angle = 15°, 60 slices/partitions, in‐plane resolution = 64 × 64, voxel size = 3 × 3 × 3 mm^3^ and GRAPPA parallel imaging in phase encoding and partition encoding direction with 2 × 3 acceleration. FMRI tasks were included in all visits, the baseline and post‐training and the neurofeedback training visits.

For the quantitative Multi‐parameter Maps (Callaghan et al., 2014; Draganski et al., 2011; Weiskopf et al., 2013), we acquired 3 spoiled multi‐echo 3D fast low angle shot (FLASH) whole‐brain volumes: (a) proton density (PD) weighted images with flip angle = 6°, TR = 23.7 ms, (b) magnetization transfer (MT) weighted images with flip‐angle = 6°, TR = 23.7 ms, a Gaussian RF pulse with 4ms duration and 220° nominal flip angle was applied 2 kHz off‐resonance before non‐selective excitation, and (c) T1‐weighted images with flip angle = 20°, TR = 18.7 ms. All volumes had a voxel size = 1 × 1 × 1 mm^3^, using a field of view (FoV) of 256 mm head–foot, 240 mm anterior–posterior, and 176 mm right–left. Details of the protocol acquisition are described in Helms and Dechent (2009) and Lutti, Hutton, Finsterbusch, Helms, and Weiskopf (2010). MPMs were acquired only during the baseline and post‐training visits.

### 2.6 Structural MRI analysis

All MPM volumes were co‐registered to correct for interscan movement using the voxel‐based quantification (VBQ) toolbox (Callaghan et al., 2014; Draganski et al., 2011). The first six echoes of each of the weighted FLASH volumes were averaged to increase signal‐to‐noise ratio and were subsequently used to calculate the MT, R1, R2*, and PD* maps as previously described (Callaghan et al., 2014; Helms and Dechent, 2009; Weiskopf et al., 2013).

Using pairwise longitudinal registration we created unbiased average MT and T1w images for the pre‐training and post‐training visits, and a volume with the Jacobian rate of change between the visits (Ashburner and Ridgway, 2013). The average MT and T1w were segmented using multispectral segmentation and the resulting grey and white matter probability maps were used to create a normalization template with DARTEL (Ashburner, 2007).

The grey, white matter, and cerebrospinal fluid (CSF) probability maps were used to calculate the intracranial volume (ICV). Caudate volume was measured using a manual segmentation procedure with MRIcro. The Striatum Atlas from the Harvard–Oxford atlas (http://www.cma.mgh.harvard.edu/fsl_atlas.html) was used to create a spatial prior for the caudate nucleus bilaterally; this was then manually edited by MP to create individual participant segmentation masks. Caudate volume was then calculated as the sum of voxels within the caudate nucleus segmentation masks bilaterally as percent of ICV.

For longitudinal voxel‐based morphometry (VBM) analysis, we masked the Jacobian rate of change for the MT volumes (Callaghan et al., 2014) with the grey matter probability volumes and normalized to MNI space using the template generated by DARTEL with modulation and applying an isotropic 6 mm FWHM Gaussian smoothing kernel. Non‐stationarity correction was applied to the whole‐brain results. Age, ICV and caudate volume (as percent ICV) were included as confounds in all analyses. Data from one participant were excluded from the analysis because of visible ringing across the brain due to large head motion; therefore, nine participants were used in this analysis.

For longitudinal VBQ analysis, the PD*, R2*, R1, and MT volumes for the pre‐ and post‐training visits were transformed into the average T1 image space by applying the deformations field created during the longitudinal registration step. The resulting images were then normalized to MNI space using the template generated by DARTEL without modulation and applying an isotropic 6 mm FWHM Gaussian smoothing kernel. The resulting images were then used to create a post‐training versus pretraining difference map which was used in the analyses. Statistical models included age, time difference between scans, ICV, and caudate volume (as percent ICV) as confounds. The results from the VBQ analyses are presented in Supporting Information.

### 2.7 Offline fMRI analysis

Statistical Parametric Mapping SPM12 (Wellcome Trust Centre for Neuroimaging, London) was used for offline preprocessing and statistical analyses of the functional and structural MRI data. The first 10 volumes were removed from the EPI time series. The images were then corrected for head‐motion with rigid‐body realignment and normalized to MNI using DARTEL without modulation and applying an isotropic 8 mm FWHM Gaussian smoothing kernel. Artrepair (http://cibsr.stanford.edu/tools/human-brain-project/artrepair-software.html) was used to identify scan‐to‐scan movement larger than 1 mm and create a regressor for the first‐level (single‐participant) models, in addition to 6 head motion parameters generated during realignment and 14 regressors modelling heart rate and breathing associated effects (Hutton et al., 2011). The images were also inspected visually for movement‐related artefacts. In total, five runs were excluded from the analysis because of the presence of artefacts due to large scan‐to‐scan movements (>2 mm) across most of the upregulate, tapping, or baseline blocks of the run. One run was further excluded because of the presence of strong RF artefacts throughout the time series.

For the statistical analyses of the neurofeedback training runs, each run was modelled separately at the first level and contrast maps were created for upregulation versus baseline. For the ROI analyses marsbar (http://marsbar.sourceforge.net/) was used to extract the contrast estimates for the target ROI mask (see section on ROI masks for details on the mask creation and Supporting Information, Figure 1 for a depiction of the mask). The ROI contrast estimates and whole‐brain contrast maps were then used in linear mixed effects models with visit and run as fixed effects and subjects treated as random effects. Age and caudate volume (as percent ICV) were also included as confounds.

For the statistical analyses of the paced tapping task, each type of run was modeled separately at the first level, for example, separate models for the pretraining and post‐training runs with and without upregulation. The contrasts for tapping versus rest were then used in a 1‐way ANOVA of differences among 3 session types: pretraining, post‐training with upregulation, and post‐training without upregulation. Age and caudate volume (as percent ICV) were included as confounds.

The functional connectivity analyses were performed using the psycho–physiological interactions (PPI) approach and using the target ROI and left putamen as seed regions. For each participant a sphere with a 6 mm radius was drawn around the voxel with the highest activation for the upregulate versus baseline contrast and nearest to the group peak for the contrast linear increase for upregulate versus baseline across visits. Masks of the target ROI and the left putamen were used such that only voxels within the masks were used to extract the seed ROI timeseries. The putamen mask was based on the AAL atlas provided by WFU Pickatlas toolbox (Maldjian, Laurienti, Kraft, & Burdette, 2003). The PPI models included the seed region time series, the condition regressor (upregulate vs baseline) and the interaction between the condition and time series regressor, the PPI regressor. The models also included 6 head motion parameters generated during realignment and 14 regressors generated modeling heart rate and breathing associated effects (Hutton et al., 2011). The first‐level contrasts for the PPI factor were then used in second‐level models to test for changes in functional connectivity from the first to the last training visit (mixed effects model with factors visit and run). Age and caudate volume (as percent ICV) were included as confounds.

Whole‐brain multiple regression analyses were performed to establish the relationship between MRI measures of functional activity, connectivity, and brain structure, with change in cognitive and motor performance after neurofeedback training. In all cases, age and caudate volume (as percent ICV) were included as confounds.

All whole‐brain and small volume correction (SVC) analyses were thresholded at voxel‐wise *p* < .001 uncorrected, cluster‐wise *p* < .05 family‐wise error (FWE) corrected for multiple comparisons. Nonstationarity correction was applied to the SVC results.

### 2.8 ROI masks

Because the target ROI was redrawn at each neurofeedback training visit using a fist clenching task as a functional localizer for the SMA, there were differences in the voxels included in the ROI across visits. This is comparable to other studies (Linden et al., 2012; Nicholson et al., 2017; Paret et al., 2014, 2016; Subramanian et al., 2011) whereby the voxels included in the calculation of the neurofeedback are different, but most relevant for neurofeedback. To determine whether there were any significant effects of visit on the size of the ROI we performed a random effects analysis using training visit as a fixed factor. This revealed that there were no significant changes in size across visits (main effect of visit *F*(3, 23.8) = 2.01, *p* = .14, increase in size from the first to the fourth training visit *t*(23.6) = 1.34, *p* = .19; mean(SE) number of voxels was 157.8(68.5), 211(65.4), 346(65.4), and 231(72.0) for the four training visits, respectively).

To further ensure that our results were focused on changes in ROI activity change and not affected by any incidental changes in ROI size, for all the analyses presented in this article, we used the group target ROI for analyses. The neurofeedback training target ROI mask was created for each participant by adding (inclusive OR) all the ROI files used in each training session together. Prior to being combined together into a group mask (exclusive OR), the ROI files were normalized and smoothed (FWHM = 8 similar to the EPI images) using DARTEL. The resulting mask was then thresholded and binarized such that all voxels from the individual participant masks were included in the group mask (voxel intensity threshold ≥ 1). A map showing the overlap of all patients' individual masks is shown on Supporting Information, Figure 1.

The mask for the striatum used for small‐volume correction in SPM12 was created using the AAL atlas provided by the WFU Pickatlas toolbox and included the putamen and caudate nucleus bilaterally.

## 3 RESULTS

**Figure 2:**
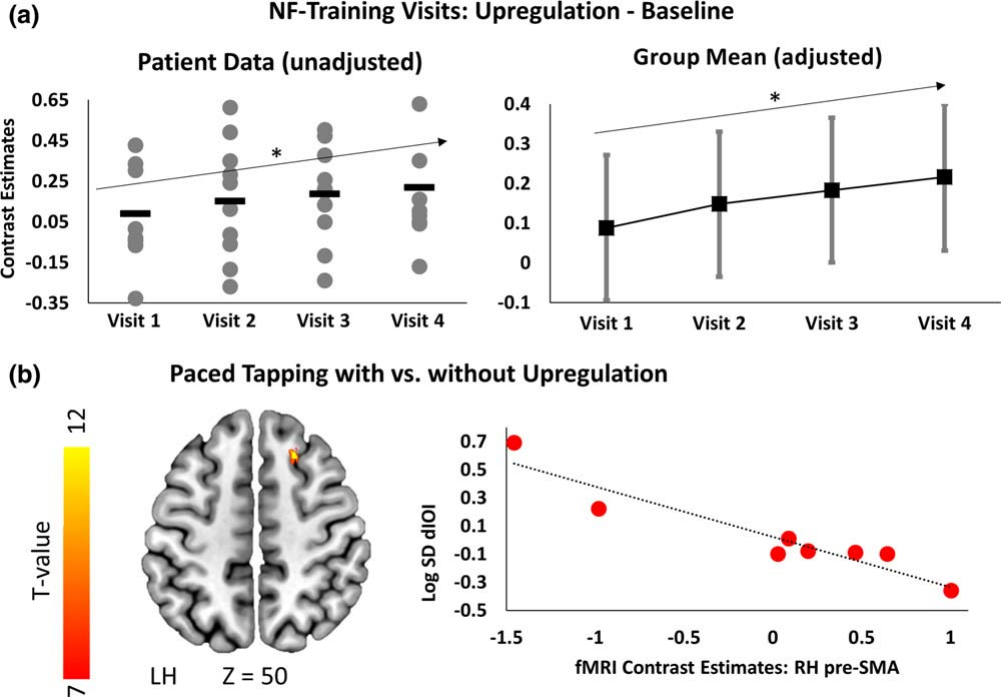
Neurofeedback training and upregulation results. (a) Plots show target ROI activation for upregulation compared to baseline for each of the four training visits. Left plot shows the unadjusted data for each participant at each visit (grey dots) and the group mean (black dash). Right plot shows adjusted means (for age and caudate volume) with 95% CI derived from the linear mixed model (black squares). In both cases, target ROI activity significantly increases from the first to the last training visit (*p < .05). (b) Superimposed on a T1 template on left are the significant voxels with the target‐ROI (small‐volume corrected, *t* value ≥ 7, *p* < .001 voxel uncorr., *p* < .05 FWE cluster corr) showing a correlation between reduced paced tapping inter‐onset interval variability (log SD dIOI) and increased activation of the right pre‐SMA during tapping with upregulation compared to tapping without upregulation after neurofeedback training. Upregulating pre‐SMA activity therefore had a beneficial effect on tapping performance (reduced variability). Scatter plot on right plots fMRI contrast estimates (tapping versus baseline with upregulation compared to without) from the right pre‐SMA cluster as a function of log SD dIOI change during tapping with versus without upregulation (red dots). Regression slope is shown in black dotted line. Pre‐SMA = pre‐supplementary motor area. *Z*‐coordinates are in MNI space

To establish whether patients were able to learn to regulate their brain activity during training, we tested for a progressive increase across time (visits and runs) of the target ROI activation when patients were asked to upregulate (compared to baseline). Using a mixed linear model with participants as a random effects factor we found a significant increase of the target ROI activation from the first to the last training visit (*t*(120) = 2.2, *p* = .03 for models both unadjusted and adjusted for age and caudate volume atrophy; Figure 2a), but no significant change from the first to the last run within visit (*t*(120) = −0.3, *p* = .76). There was also no significant run by visit interaction (*F*(9, 120) = 0.77, *p* = .64). Although there was a significant, linear, mean increase across visits, there was evident variability in signal change across visits between participants (Figure 2a). Similarly, the voxels within the target ROI that showed a significant effect of training were also variable between participants, suggesting that there were differences within the group in terms of training effects.

Although during training, the neurofeedback presented was based on the average signal from the target ROI, it is likely that only a subpart of the selected region would be engaged by the participants. To identify the exact voxels that showed a change across training, we performed fixed effects analyses (at the single‐participant level) characterizing the increase in BOLD signal within the target‐ROI during upregulation across training visits. In Supporting Information, Figure 3a, we show the significant clusters within the target‐ROI for each of the participants (at threshold *p* < .001 voxel uncorrected, *p* < .05 cluster FWE‐corrected), and plot the contrast estimates for upregulation vs baseline across all visits from the significant clusters, which are more representative of the participants' training progress (Supporting Information, Figure 3b). Our group of HD patients was therefore able to learn to regulate the activity of the target region during training, although there was variability within the group. We then asked what the effects of the learnt volitional regulation were on the activity of the target ROI and motor performance, while they were performing a simple motor task.

On separate visits within 2 weeks after completion of the neurofeedback training, patients performed in the scanner paced tapping with the left index finger with and without volitional upregulation of the target ROI. Paced tapping is a simple motor task that engages the SMA and also ensures that motor performance, such as number of taps and speed, is comparable across the different sessions. Therefore, it allows us to observe changes in motor network activity due to upregulation, unconfounded by any gross differences in motor performance, such as differences in number of taps. Indeed there were no significant differences in the number of taps across the three sessions (*F*(2, 14) = 1.27, *p* = .31; adjusted mean(SE) number of taps was 35.10(0.7), 35.05(0.7), and 36.00(0.7) for pretraining visits and post‐training without and with upregulation, respectively. Also see Supporting Information). As a subtle measure of motor performance for these analyses, we used variability in deviation from the paced tapping rhythm (log SD dIOI). This is a sensitive marker of HD motor impairment and progression measuring the capacity of patients to maintain a rhythm (Bechtel et al., 2010), whereby the larger the variability, the greater the motor impairment. To determine whether patients learned to control their activity after training, we compared tapping with and without upregulation. The comparisons between the pretraining and post‐training visits are presented in the Supporting Information for completeness.

Using a repeated measures model of tapping performance with the presence or absence of upregulation as a factor, plus covariates age and caudate volume, we found no significant upregulation effect upon log SD dIOI (*F*(1, 5) = 0.064, *p* = .81; adjusted mean(SE) = 3.98(0.92) and 4.00(0.13). Only data from 8 subjects were included in this analysis, refer to the Participants section for more details). In analogous statistical models contrasting fMRI activation during tapping with upregulation versus without, we also did not find any significant differences either across the whole‐brain or within the target ROI (at threshold *p* < .001 voxel uncorrected, *p* < .05 cluster FWE‐corrected). Based on our results, therefore, patients did not show substantial evidence of learnt control at a group level, while performing a simple motor task.

To examine whether there were differences within the group in the patients' ability to upregulate the fMRI activation of the target ROI after training, we performed regression analyses using tapping performance as a predictor. We hypothesized that improved performance in the tapping task after training would correlate with ability to upregulate the target ROI activation. Indeed, regression analyses using log SD dIOI as a performance marker showed that lower variability, that is, better performance, predicted increased fMRI activation within the target ROI, and other motor areas during tapping with upregulation compared to without. More specifically, significant clusters were within the target ROI (small‐volume‐corrected results within the target ROI; pre‐SMA cluster peak MNI: 21 24 50, cluster size = 37 voxels, cluster FWE‐corrected *p* < .001; Figure 2b), and in RH dorsal pre‐SMA (cluster peak MNI: 26 8 50, cluster size = 148 voxels, cluster FWE‐corrected *p* = .003) and RH cerebellum (cluster peak MNI: 40 −68 −32, cluster size = 121 voxels, cluster FWE‐corrected *p* = .010). Patients therefore differed in the degree to which they were able to volitionally upregulate the activity within the target region in the absence of neurofeedback while performing a simple motor task. Importantly, patients who succeeded in volitionally upregulating their target ROI activity after training had better task performance, suggesting that success at learnt control of the target ROI activation can lead to improvement in motor performance.

**Figure 3:**
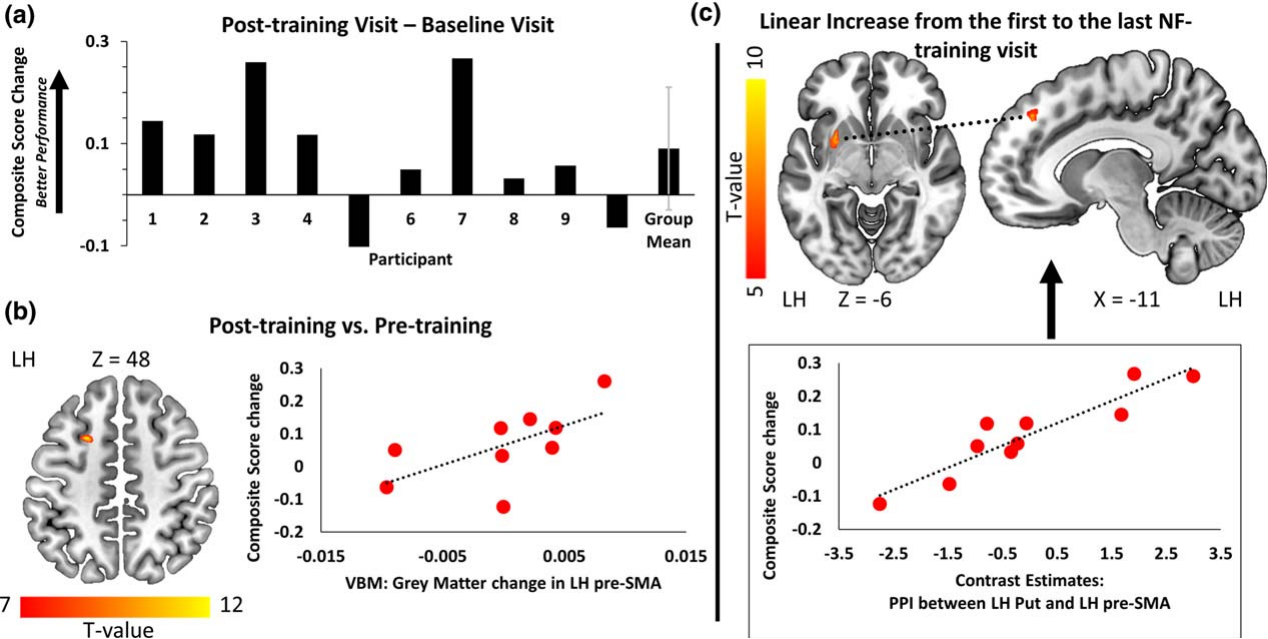
Change in performance after training and correlation with functional and structural changes. (a) Change in the composite score after neurofeedback training compared to before for each patient and the group mean (SD) (last column). Positive scores along the *y*‐axis indicate that patients performed better overall after training compared to before. (b) VBM: overlaid on T1 template on left are significant voxels (*t* value ≥ 7, *p* < .001 voxel uncorr, *p* < .05 FWE cluster corr) showing a correlation between improvement in the composite score and increase in grey matter volume in the left pre‐SMA after training compared to before. On right contrast estimates from the left pre‐SMA cluster are plotted as a function of composite score change. Regression slope shown in black dotted line. (c) Top left: voxels in the left putamen showing significant correlation between improvement in the composite score after training and increased activation from the first to the last neurofeedback training visit (SVC within the striatum bilaterally, *t* value ≥ 5, *p* < .001 voxel uncorr, *p* < .05 FWE cluster corr). Top right: voxels within the target ROI showing significant correlation between improvement in the composite score and increased functional connectivity (PPI) with the left putamen from the first to the last training visit (SVC within the target ROI, *t* value ≥ 5, *p* < .001 voxel uncorr, *p* < .05 FWE cluster corr). Results are overlaid on a T1 template in MNI space. Scatter plot at the bottom shows the PPI contrast estimates from the left pre‐SMA cluster (shown on top right) as a function of composite score change. Regression line shown in black dotted line. VBM = voxel‐based morphometry; Pre‐SMA = pre‐supplementary motor area; SVC = small volume correction; PPI = psychophysiological interactions

To evaluate whether the effects of neurofeedback training extended beyond the period when participants were engaged in volitional upregulation of their brain activity, we compared patient performance before and after training in a set of untrained, cognitive and motor measures sensitive to HD progression. We did not have a priori hypotheses about which specific measures would improve significantly following the training, but were interested in whether neurofeedback training would have a benefit on cognitive and motor function overall. As a measure of cognitive and motor capacity we therefore employed a previously used composite score approach (Klöppel et al., 2015) that comprised of a priori selected tasks from the Track‐HD battery, that were unrelated to the neurofeedback training and are sensitive to HD progression (Tabrizi et al., 2009, 2012, 2011, 2013) (see Methods for more details). To account for short‐term practice related effects and stabilize participant performance, all assessments were performed twice on separate visits prior to the onset of the training; the second assessment was the baseline visit and compared against the assessment after training. Patients were not instructed to upregulate their brain activity during these tests. The calculated composite score at the baseline visit was sensitive to disease pathology and stage. Patients with low scores had smaller caudate volume, an accurate measure of disease pathology (Spearman's *r* = .82, *p* = .006), and high normalized CAG age product (Ross et al., 2014) (CAP score), an estimate of disease stage (Spearman's *r* = −.80, *p* = .010). All correlations were adjusted for age, but results were significant without adjustment as well (all *p* < .02). The composite score after training was higher (better) than the baseline visit measure in 8 out 10 patients, although not significant (group mean (SD) = 0.70 (0.68) and 0.76 (0.65) for the baseline and post‐training visits, respectively; paired *t*‐test *t*(9) = 2.18, *p* = .057; Figure 3a). The change across all seven measures that comprised the composite score is presented in Supporting Information, Figure 4 for completeness. However, because we did not have an a priori hypothesis about which of the individual measures should show improvement following neurofeedback training, we did not perform any post‐hoc statistical analysis and the data presented are only descriptive. Our results therefore suggest that although all patients successfully learnt to upregulate activity within the target ROI during neurofeedback training, not all of them improved in measures of cognitive and motor function.

To identify the changes in brain structure and function that correlate with the improvement in cognitive and motor performance that we observed, we used linear regression to examine the structural and functional MRI changes during and following training that correlated with improved function. Changes at the group level (without correlation with performance) are presented in the Supporting Information for completeness. In terms of changes in brain structure, we performed VBM and show that improvement in the composite score after training correlated significantly with an increase in left pre‐SMA grey matter (GM) volume after training (cluster peak MNI: −22 10 48, cluster size = 54 voxels, cluster FWE‐corrected *p* = .009; located by the left border of the functionally defined target ROI mask; Figure 3b) and in the right inferior frontal gyrus (RIFG; cluster peak MNI: 58 16 8, cluster size = 54 voxels, cluster FWE‐corrected *p* = .001).

To examine changes in brain function during training, we performed random effects linear regression analyses examining the relationship between change in the composite score and change in the fMRI signal levels from the first to the last training visit. Contrary to expectation, improvement in the composite score did not predict increasing activation from the first to the last training visit within the target ROI (regression with ROI estimates (F1, 9) = 3.35, *p* = .104; as well as no significant clusters using small‐volume correction within the target ROI), but instead in the left putamen (small‐volume‐corrected results within the striatum bilaterally; cluster peak MNI: −27 4 −8, cluster size = 111 voxels, cluster FWE‐corrected *p* < .001; Figure 3c). It also predicted decreasing activation from the first to the last training visit in the right supramarginal gyrus (SMG; cluster peak MNI: 56 −18 32, cluster size = 656 voxels, cluster FWE‐corrected *p* < .001). Although the left putamen was not directly targeted during neurofeedback training, its activation during upregulation could have been modulated indirectly via afferent connections to the target ROI. In this case, we would expect to also see a correlation between improved performance and increase in functional connectivity between the target‐ROI and the left putamen during the training.

We used a psycho–physiological interactions approach (Friston et al., 1997) to test for changes in functional connectivity from the first to the last training visit between the target ROI and the left putamen cluster that was previously shown to correlate with improved performance. Change in the composite score after training was then used as a regressor to identify the functional connectivity changes that correlate with improvement in behavior. Our results are consistent with the notion of training a wider motor network. Using as seed ROI the left putamen cluster we found that improved performance predicted increase in functional connectivity with the target ROI (small‐volume‐corrected results within the target ROI; pre‐SMA cluster peak MNI: −12 40 40, cluster size = 41 voxels, cluster FWE‐corrected *p* = .010; Figure 3c). Using the target ROI as the seed region we found that improved performance also predicted increased connectivity between the target ROI and the right cerebellum from the first to the last training visit (cluster peak MNI: 26 −78 −52, size = 249 voxels, cluster FWE‐corrected *p* < .001). The increased connectivity between the left putamen and the pre‐SMA cluster significantly correlated with the increase in pre‐SMA GM volume (Spearman's *r* = .78, *p* = .017), but not with increased left putamen activity (Spearman's *r* = .16, *p* = .66) or the increase in target ROI activity during the training (Spearman's *r* = −.21, *p* = .56). The changes in left pre‐SMA GM volume are therefore associated with training‐related changes in functional connectivity between the left pre‐SMA and putamen. The results from the regression analyses provide a link between training‐related structural and functional plasticity and improvement in overall cognitive and motor performance. They also suggest that neurofeedback training can induce plasticity in HD patients despite the presence of neurodegeneration and that the effects of training in a single region can extend to corticostriatal circuits implicated in the disease pathology.

## 4 DISCUSSION

In this study, we have presented preliminary evidence to suggest that neurofeedback training is feasible in HD, and may induce disease‐relevant neuroplasticity with potentially beneficial effects on cognitive and motor function. We did not include a control group, as the main aim of the study was to show proof‐of‐concept, feasibility, and provide important data to inform future trials. This approach is consistent with other proof‐of‐concept neurofeedback training studies (Corlier et al., 2016; Nicholson et al., 2017). In terms of our primary aim, we have shown that HD patients can be trained to regulate their own brain activity using neurofeedback training. In addition, the results from the regression analyses identify a statistically significant link between training‐related plasticity and improvement in performance in patients with a neurodegenerative condition. It is notable that in a disease such as HD, which is characterized by progressive atrophy, we also observed increased GM volume following the training in the participants that improved in performance. To our knowledge, this is the first time that an increase in GM volume has been observed after neurofeedback training, which is possibly because our training protocol, including 3–4 training visits per participant on a weekly/bi‐weekly basis, was more intense than those previously reported.

To further understand the potential benefits of upregulating one's own brain activity, we asked participants after the training to perform a simple motor task (paced finger tapping) in the scanner, while they attempted to upregulate the activity of their target ROI in the absence of neurofeedback. We found that participants that were able to upregulate their brain activity, while tapping, performed the task better, than those who could not upregulate their activity. This provides additional evidence that self‐regulation of one's own brain activity, can have beneficial effects on performance. It is an interesting question, why some participants were able to upregulate and others not. Because of the relatively small sample size for this feasibility study, it is not possible to draw any strong conclusions. However, even in studies with healthy young adults, some participants are not able to learn to self‐regulate (Chiew, LaConte, & Graham, 2012; Scharnowski et al., 2012). The reasons for this difference, between learners and nonlearners, remain unclear.

In this study, we selected the SMA as a target ROI for neurofeedback training because of its role in motor control and HD symptoms. SMA activation and SMA–striatal connectivity correlate with disease progression and symptoms in HD (Bohanna et al., 2011; Klöppel et al., 2015; McColgan et al., 2015; Novak et al., 2015). We found that patients learned to increase their SMA activation across the training visits and that performance improved in those patients who were able to upregulate their SMA activity while performing a tapping task after training. However, improvement in overall cognitive and motor performance in untrained tasks was not related to change in SMA (target ROI) activity, but rather change in left putamen activity and the connectivity between the pre‐SMA and the left putamen. Efferent connections and striatal activity are engaged during neurofeedback training (Emmert et al., 2016; Haller et al., 2013; Koush et al., 2013; Nicholson et al., 2017; Ruiz et al., 2013; Scharnowski et al., 2012; Zhang et al., 2015; Zotev et al., 2011), reflecting cognitive processes that are also involved in neurofeedback training, for example, change in attention and reward processing. In our study, the engagement of the left putamen could reflect the use of motor imagery and reinforcement learning during neurofeedback training, stimulating SMA–striatal connectivity. The fact that strengthening of cortico‐striatal connectivity correlated with improvement in behavior further highlights its role in HD pathology and suggests that stimulating cortico‐striatal connectivity might be more appropriate as a target for neurofeedback training compared to SMA training. Our findings are consistent with recent claims that changes in brain networks during neurofeedback training mediate improvements in behavioral performance (Zhang et al., 2015).

Because of the absence of a control group, it is difficult to establish whether the observed changes in brain function and structure, as well as cognitive and motor performance, came about because of the training or because of placebo or other training‐unrelated effects. However, we believe it is unlikely that the changes that were observed in cognitive and motor performance could be attributed to practice effects. Although cognitive and motor scores could be improved because of practice alone, most of the improvement takes place between the first and second repetition (screening and baseline visits) (Stout et al., 2014). In our case, the composite score change that we reported was between the second and third repetition (baseline and post‐training visits). Furthermore, practice effects of the cognitive and q‐motor tasks cannot fully explain the correlation between the change in the composite score and the increase in putamen activity and connectivity from the first to the last training visit. Patients did not perform the cognitive and motor tasks during the 3–4 training visits, but in separate visits before and after the training that were at least 1 month apart. Therefore it is unlikely that an increase in cortico‐striatal connectivity from the first to the last training visit, would relate to simple task practice effects, unrelated to the training.

It is however possible that the practice of motor imagery, which occurred during neurofeedback training, could be responsible for the observed changes in brain function and structure as well as improvements in cognitive and motor performance. Because both motor imagery and neurofeedback were used concurrently, the presentation of neurofeedback could have contributed to improvement in motor imagery and vice versa. Similar to other proof‐of‐concept neurofeedback training studies (Corlier et al., 2016; Nicholson et al., 2017), it was beyond the scope of this study to disentangle the contribution of the cognitive strategy involved in the training (motor imagery in this case) from the effect of receiving neurofeedback. Previous studies have used neurofeedback training and included control groups that were practicing motor imagery alone, without neurofeedback. They have all shown that motor imagery alone is not as effective as neurofeedback in increasing brain activity during the training and improving behavior after training (Sepulveda et al., 2016; Subramanian et al., 2011; Yoo, Lee, O'leary, Panych, & Jolesz, 2008). However, the small size of these studies, and the fact that in some cases the participants were not blinded to their group allocation (Subramanian et al., 2011), makes it equally difficult to draw any firm conclusions. It is possible that differences in performance could be attributed to differences in motivation and placebo effects. To answer this question in a definitive way, future, randomized and single‐ or double‐ blinded trials are required that would include a sham‐neurofeedback group. Our study provides important data that can be used to design such future trials.

The neurofeedback training protocol adopted in our study included shaping, whereby the scale of reward presented to the participants during each block was dependent on their performance in the previous block. Shaping has been previously used in other neurofeedback training studies (De Charms et al., 2004; Linden et al., 2012), with different studies using different ways of implementing shaping. Linden et al. (2012) implemented shaping by choosing different voxels across the brain as targets for neurofeedback after each run. De Charms et al. (2004) followed a different approach, whereby the target level of activation was updated after each block using a 3 up 1 down approach. In this study, we chose to update the target level of activation after each block using the maximum PSC from the previous block, because we aimed at keeping the participants motivated throughout the run. By adjusting the target level for every block participants constantly applied effort in order to receive positive feedback, while also ensuring that they did not become demotivated if they were underperfoming. This was particularly important for our patient group, which is highly anxious and could become distressed in the scanner if, for example, they underperformed consistently. In this way, we rewarded positive performance even if it was low. At present there is no evidence to determine the optimal shaping protocol for real‐time fMRI neurofeedback training. To answer this question, future studies would need to be designed that can compare different shaping approaches. In this study, the fact that our participants learned to increase their target‐ROI activation is evidence in support of the fact that our training protocol was successful.

## 5 CONCLUSION

We have shown that HD patients can learn to regulate their own brain activity using neurofeedback training. Importantly, we were able to identify the functional and structural changes that occurred during neurofeedback training and which correlated with cognitive and motor improvement in a set of (untrained) measures sensitive to disease progression. Our data suggest that functional connectivity between the SMA and the left putamen may be a promising target for neurofeedback training. Our results can inform the design of future larger, randomized and controlled studies, which are now required to provide stronger evidence on the effectiveness and benefits of this approach. Because it is noninvasive, neurofeedback training could be used preventatively or as adjunct treatment (Linden, 2012) to other disease‐modifying therapies and restore function in HD and other neurodegenerative diseases.

## 6 DATA AVAILABILITY

All relevant data are available from the authors.

## CONTRIBUTIONS

M.P., N.W., G.R., and S.J.T designed the study. M.P. performed neuropsychological testing, MRI data acquisition, data processing, and statistical analysis. D.L. supported the statistical analysis. R.R. supported the processing of the Q‐Motor data. M.P. and G.R. prepared the manuscript. All authors discussed results and commented on the manuscript.

## COMPETING FINANCIAL INTERESTS

The Wellcome Trust Centre for Neuroimaging has an institutional research agreement with and receives support from Siemens Healthcare.

## Supporting information

Additional Supporting Information may be found online in the supporting information tab for this article.

Supporting Information
