# Supplementary material for: Stimulating neural plasticity with real‐time fMRI neurofeedback in Huntington's disease: A proof of concept study
Source: Hum Brain Mapp. 2017 Dec 13;39(3):1339–53. doi: 10.1002/hbm.23921 (PMC5838530; doi:10.1002/hbm.23921)
Supplement: Supplementary file 1 — Supporting Information [file HBM-39-1339-s001.docx]

Stimulating neural plasticity with real-time fMRI neurofeedback in Huntington’s disease: a proof of concept study

Marina Papoutsi (1), Nikolaus Weiskopf (2, 3), Douglas Langbehn (4), Ralf Reilmann (5, 6), Geraint Rees (3, 7), Sarah J Tabrizi (1)

(1) Huntington’s disease centre, Institute of Neurology, University College London, UK, (2) Max Planck Institute for Human Cognitive and Brain Sciences, Leipzig, Germany, (3) Wellcome Trust Centre for Neuroimaging, Institute of Neurology, University College London, UK, (4) Carver College of Medicine, University of Iowa, USA, (5) George Huntington Institute and Dept. of Radiology University of Muenster, Germany, (6) Section for Neurodegeneration and Hertie Institute for Clinical Brain Research, University of Tuebingen, Germany, (7) Institute of Cognitive Neuroscience, University College London, UK

**Supplementary Materials**


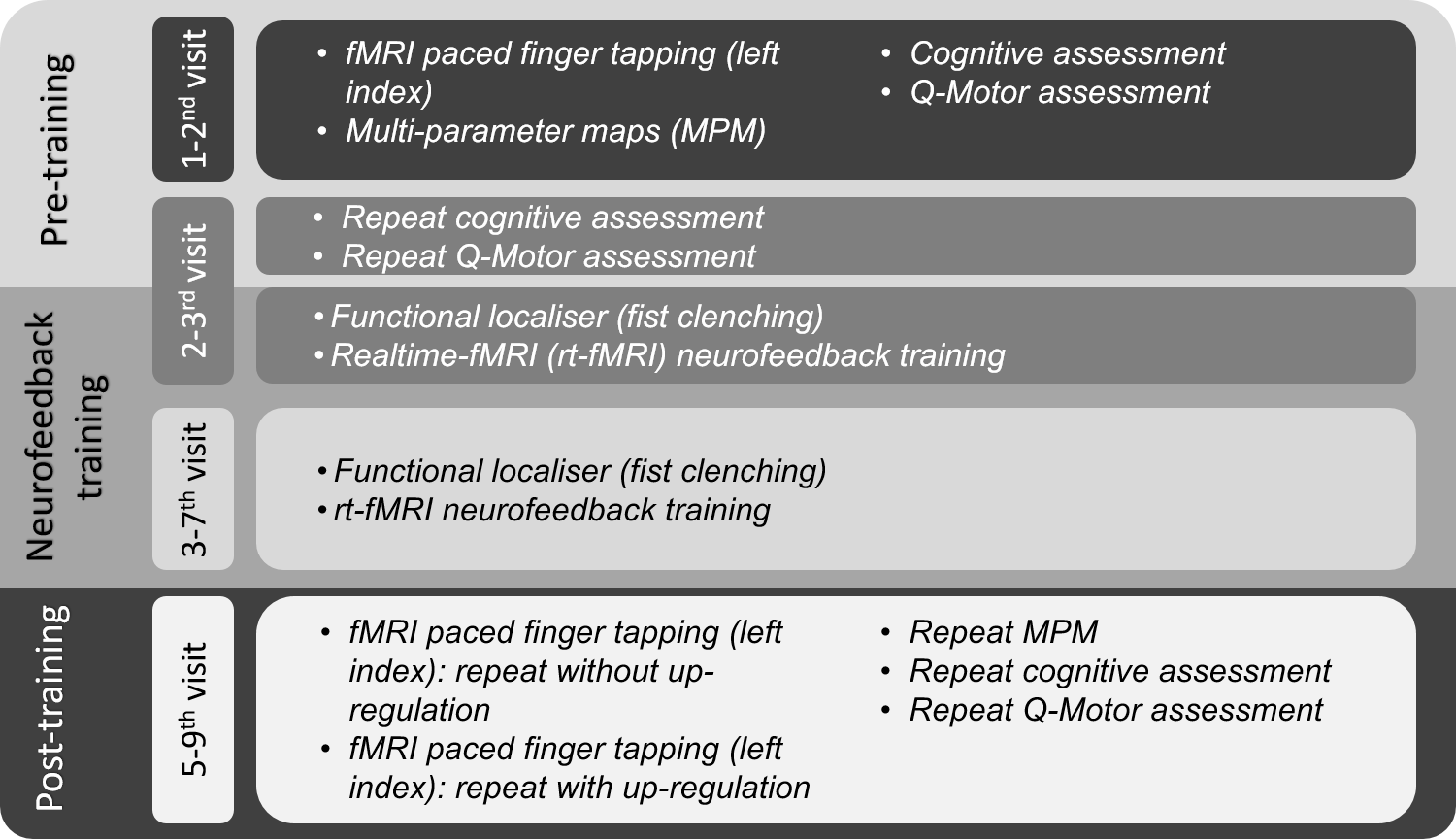


**Supplementary Diagram 1:** Study Structure. Structural and functional MRI, cognitive and Q-Motor assessments took place prior to the first neurofeedback training MRI session. Cognitive and Q-Motor assessments were performed twice before the first neurofeedback training session, with the 2nd assessment used as baseline to minimize practice effects. Patients completed 3-4 neurofeedback training visits on separate days. After the last neurofeedback training visit participants repeated all baseline assessments during a follow-up visit on a separate day. A variation of the paced tapping task with concurrent upregulation of the target ROI was also introduced at the post-training visit.

**
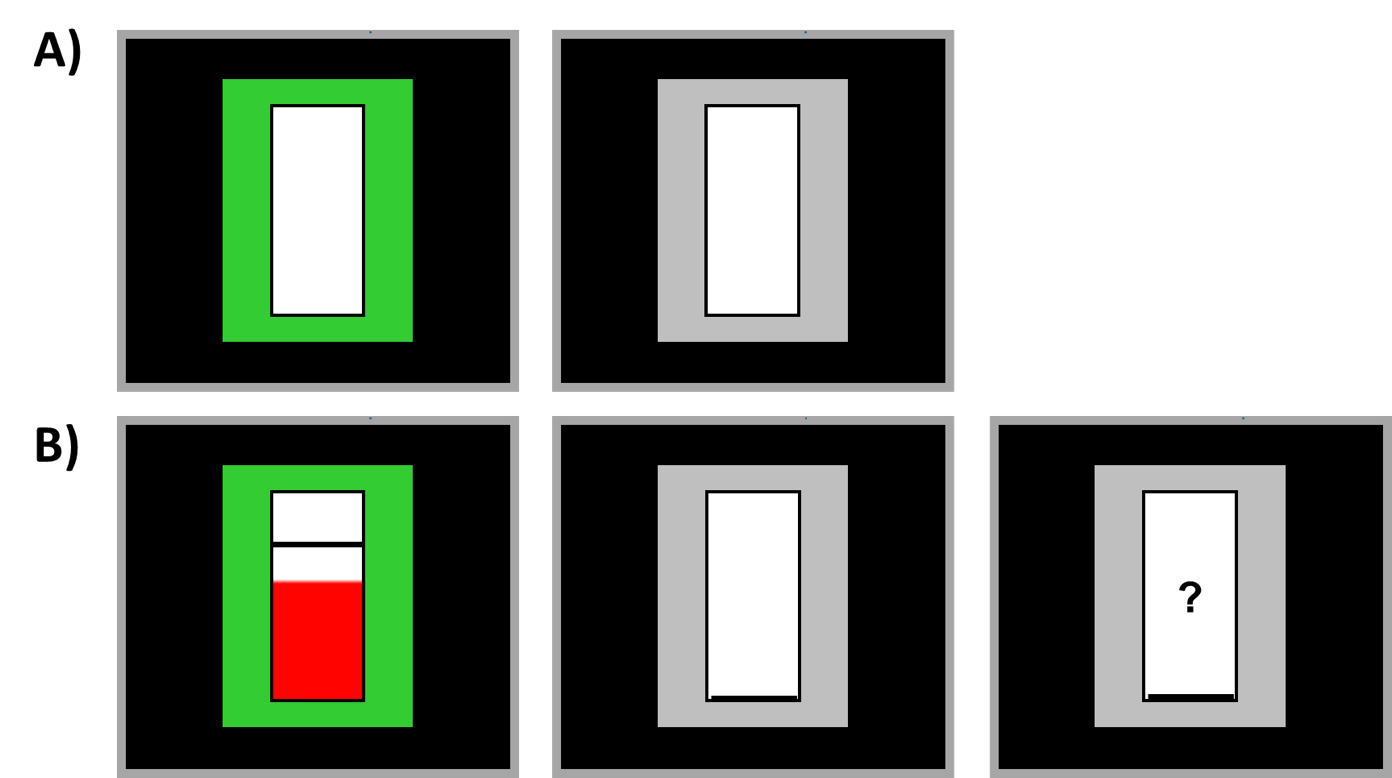

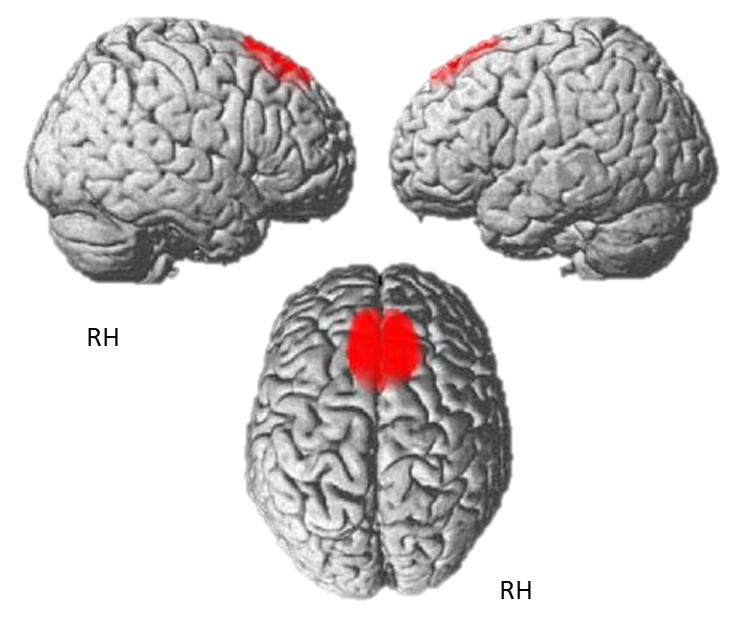
Supplementary Figure 1:** Overlay of the target ROI used for neurofeedback training combined across all participants. RH = right hemisphere.

**Supplementary Figure 2:** (A) Visual presentation for the paced tapping and fist clenching tasks and (B) the neurofeedback training runs. Visual presentation was kept consistent across the different tasks (green frame for “active”, grey frame for “passive” blocks) to condition the patients to act during the “active” blocks and rest during the “passive” blocks and therefore facilitate greater task compliance, especially during the neurofeedback training runs. The black line was set high during the neurofeedback blocks and low during the baseline blocks and acted as an additional visual cue to encourage the participants to upregulate only during the active blocks.

**
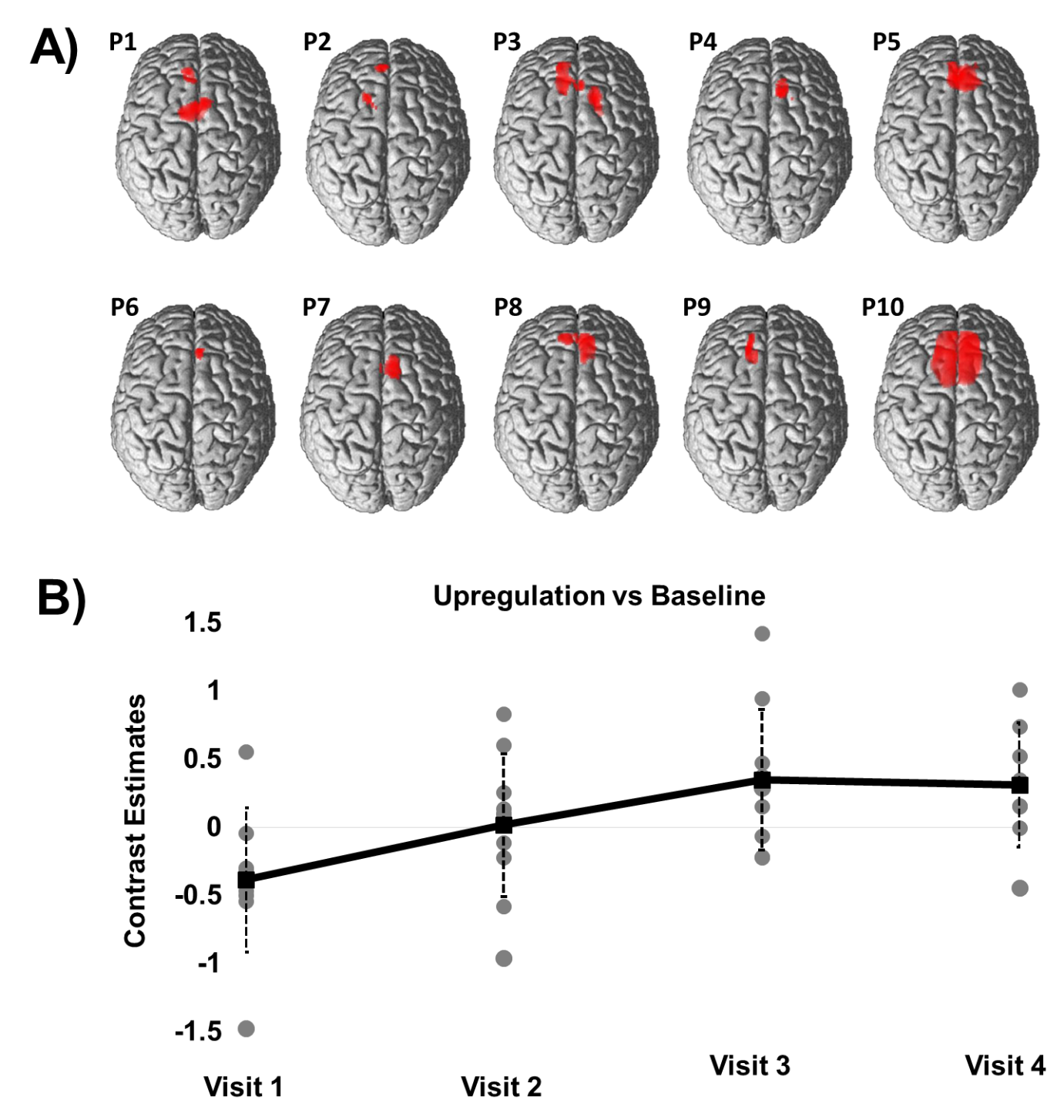
Supplementary Figure 3:** (A) Fixed effects analyses were used at the single-subject level to identify for each participant the clusters within the neurofeedback target ROI that showed a significant change in brain activity over the course of the training. All participants had significant clusters within the target ROI that showed a training effect. The clusters shown in red show a significant increase in BOLD signal during upregulation compared to baseline from the first to the last training visit for each participant. Results are thresholded at p < 0.001 voxel uncorrected, p < 0.05 FWE cluster corrected only within the area of the neurofeedback target ROI (small-volume corrected). (B) The estimates for the contrast upregulation vs baseline were extracted from each of the clusters shown in (A) for all runs. The BOLD signal increases during upregulation compared to baseline during the course of the training (also see figure 1A and 1B in the main article). The black continuous line represents the group mean, the error bars are the group standard deviation. The grey dots are the mean visit values for each participant.


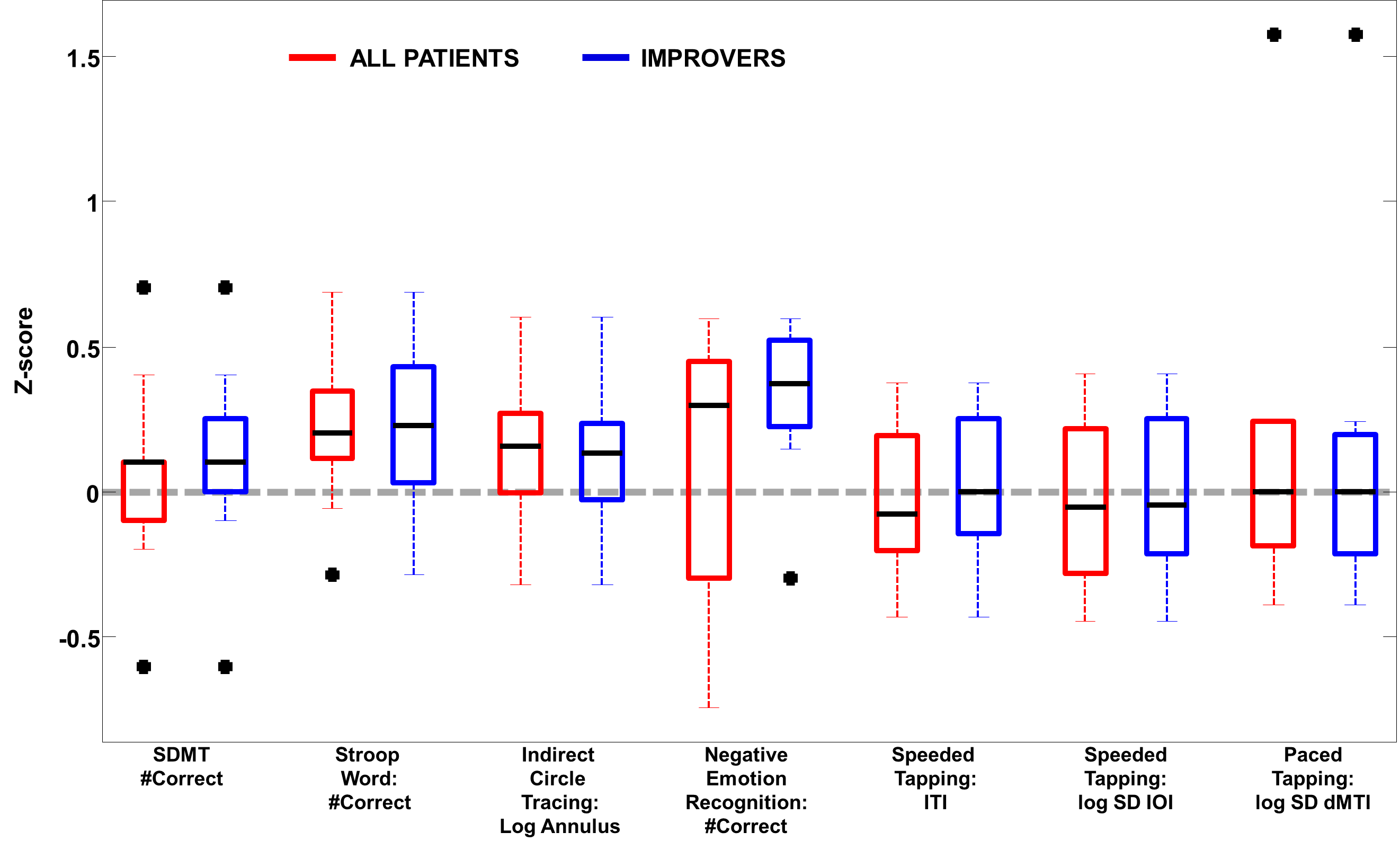


**Supplementary Figure 4:** Shown is the change in the seven measures that comprise the composite score after the neurofeedback training compared to baseline across all 10 participants (red) and the 8 improvers only (blue). A positive value on the y-axis represents improvement in performance after training compared to baseline. The grey dotted line shows zero, which represents no change between the visits. The central black line in the boxes shows the group median change, the box edges are the 25^th^ and 75^th^ percentiles, the whiskers extend to the most extreme datapoints, the outliers are shown as black dots. We did not have an a-priori hypothesis about which score would change because of neurofeedback training, so used a composite score to test for change overall instead of testing every measure separately. The cognitive measurements that comprised the composite score were: number correct for Stroop Word Reading only, number correct for Symbol Digit Modalities Test (SDMT), annulus length for Indirect Circle Tracing (log transformed) and number correct for negative Emotion Recognition. The Q-Motor measurements included were: inter-tap interval (ITI) and standard deviation of inter-onset interval (log transformed; log SD IOI) during speeded tapping with the left (non-dominant) index finger, and standard deviation of mid-tap interval deviation from target rhythm (log transformed; log SD dMTI) for paced tapping with left index finger at 1.8Hz. All of these measure were selected a-priori from the Track-HD battery, because of their sensitivity to disease progression^2^.

***Structural Plasticity: VBM & VBQ Results***

At the group level (without including performance as a regressor), similar to previous studies on training-related structural plasticity^3,4^ we observed significant changes in structural and functional MRI in regions of the motor network. These results only show the changes that are common across all participants after training, but not the changes that correlate with improvement in cognitive and motor performance, and were therefore not of interest. These results from the regression analyses are presented in the main manuscript. The group results are presented here for completeness.

In terms of structural changes, the VBM analyses examining the group average effects showed increased grey matter (GM) volume after training compared to before within the RH Middle Frontal Gyrus (cluster peak MNI: 34 36 27, size = 585 voxels, FWE-corr p < 0.001; Supplementary Figure 5A). The VBQ analysis looks at changes in MR parameters related to macromolecular content. The results showed increase in PD* values in the RH precentral gyrus (preCG; cluster peak MNI: 33 -10 52, size = 272 voxels, FWE-corr p = 0.006), as well as increased MT values in the cerebellum bilaterally (cluster left peak MNI: -34 -66 -27, size =263 voxels, FWE-corr p = 0.030, and right peak MNI 8 -72 -34, size = 270 voxels, FWE-corr p = 0.026) and left putamen (small-volume correction using bilateral striatum mask; cluster peak MNI: -21 10 -8, size = 86 voxels, FWE-corr p < 0.001; supplementary Figure 5B and 6C). There were no other positive or negative results or significant associations with performance.


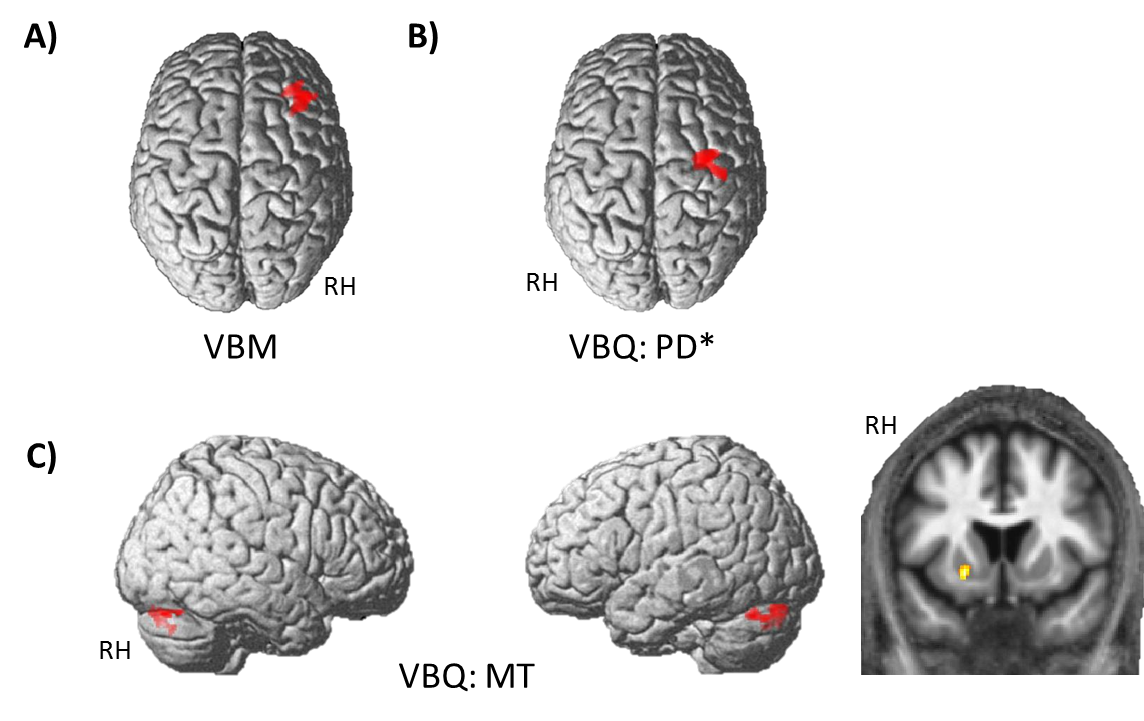


**Supplementary Figure 5:** Structural plasticity following neurofeedback training. (A) VBM whole-brain results showing significant grey matter volume increase in the RH middle frontal gyrus after training compared to before (p<0.001 voxel uncorr., p<0.05 FWE cluster corr). (B) VBQ whole-brain results showing increased PD* signal in the grey matter of the RH precentral gyrus (p<0.001 voxel uncorr., p<0.05 FWE cluster corr). (C) Results showing voxels with increased MT signal in the cerebellum bilaterally (whole-brain p<0.001 voxel uncorr., p<0.05 FWE cluster corr ) and the LH putamen (small volume correction within the striatum bilaterally p<0.001 voxel uncorr., p<0.05 FWE cluster corr).

***Functional Plasticity: Paced Tapping fMRI Task***

A paced tapping task with the left index finger (non-dominant hand in all cases) was used to examine changes in activity within the motor network after neurofeedback training with and without concurrent upregulation. We selected a paced tapping task, because it is a validated HD biomarker and also because motor performance, such as number of taps and speed of tapping, can be controlled and kept similar between the different conditions. Thus, differences in brain activity between the different conditions could not be attributed to gross differences in performance. To test for differences between the 3 conditions of interest: paced tapping at baseline (no upregulation), paced tapping after training without upregulation and paced tapping after training with upregulation, we used a mixed model with factor condition and confounds caudate volume (as percent ICV) and age. For the analysis of the tapping performance, the first five pacing tones per block were ignored to allow for the response to stabilize.

As expected there was no significant difference in number of taps across the 3 visits: (F(2, 14) = 1.27, p = 0.31; adjusted mean(SE) number of taps was 35.10(0.7), 35.05(0.7) and 36.00(0.7) for pre-training visits and post-training without and with upregulation respectively). Variability in deviation from the paced tapping rhythm (log SD dIOI), a more subtle measure of motor performance, has been previously shown to be a reliable marker of disease progression^5–8^. There were also no significant differences between the 3 visit types for this measure: (F(2,14) = 0.06, p = 0.94; adjusted mean(SE) log SD dIOI was 4.01(1), 4.00 (1) and 4.00(1) for pre-training visits and post-training without and with upregulation respectively).

In terms of brain activity, at the group level comparison of paced tapping before training (no upregulation) and after training without upregulation did not reveal any significant changes. However, regression analysis using variability in dIOI (log SD dIOI) as a measure performance showed that lower variability (i.e. better performance) correlated with higher activation in the RH postcentral gyrus (cluster peak MNI: 32 -38 64, size = 316 voxels, FWE-corr p < 0.001) after training compared to before (Supplementary Figure 6A). There were no significant results for the opposite contrast.

Group-level comparison of paced tapping before training (no upregulation) and after training with upregulation did not reveal any significant changes in fMRI activation. However, regression analysis with log SD dIOI shows that lower variability (better performance) during tapping with upregulation correlated with higher activity within the target ROI (small-volume-corrected results within target ROI. RH pre-SMA cluster peak MNI: 22 28 50, size = 27 voxels, FWE-corr p < 0.001; Supplementary Figure 6B), RH precuneus (cluster peak MNI: 12 -56 27, size = 206 voxels, FWE-corr p < 0.001) and LH superior occipital gyrus (cluster peak MNI: -14 -88 14, size = 91 voxels, FWE-corr p = 0.047). On the contrary, increased variability predicted increased activity in the RH inferior frontal gyrus (pars orbitalis; MNI: 30 24 -8) suggesting that it has a negative effect on performance.

Group comparison of the paced tapping runs after training with and without upregulation revealed a significant decrease in activation during tapping with upregulation compared to without in a cluster located on the RH medial superior frontal gyrus (SFG; cluster peak MNI: 0 56 26, size = 1226 voxels, FWE-corr p < 0.001) and the LH rolandic operculum (cluster peak MNI: -39 -32 21, size = 659 voxels, FWE-corr p = 0.005). The medial SFG cluster falls within the target ROI (small volume corrected results within the target ROI; cluster peak MNI: 2 45 40, size = 478, FWE-corr p = 0.001), however it is not located within the pre-SMA and is more frontal than the cluster shown in Figure 2B (main article) and which correlates with improved performance.

It is notable that after training, better motor performance during tapping without upregulation compared to before training is associated with increased activity of the postcentral gyrus, whereas better motor performance during tapping with upregulation compared to before training is associated with increased activity within the target ROI (amongst other regions). This dissociation suggests that (a) participants performed the tasks with and without upregulation differently and according to the instructions and were able to control when they upregulate and when not, and (b) that neural changes after neurofeedback training were not limited only to cases where participants explicitly upregulated and to only the trained region.


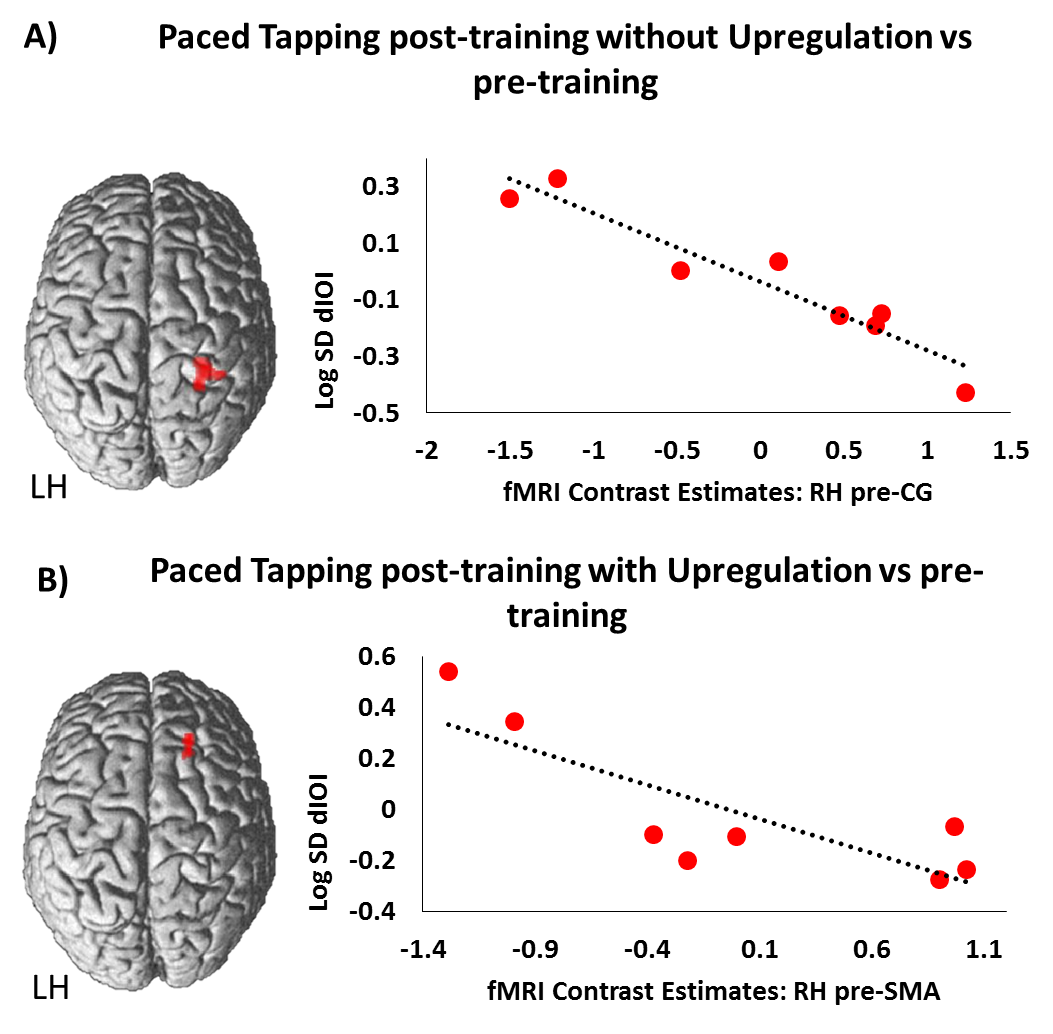


**Supplementary Figure 6:** Regression with change in paced tapping performance after neurofeedback training compared to before. (A) Whole-brain results showing voxels in the right preCG where higher activation correlated with lower variability (log SD dIOI; better performance) during paced tapping without upregulation after training compared to before (p<0.001 voxel uncorr., p<0.05 FWE cluster corr). Scatter plot shows contrast estimates from the preCG cluster (red dots) and the model regression line (black dotted line). (B) Results showing voxels within the target ROI whereby increased activation correlated with lower variability (log SD dIOI) during paced tapping with upregulation (after training) compared to before (small volume correction within the target ROI p<0.001 voxel uncorr., p<0.05 FWE cluster corr). Scatter plot shows contrast estimates from the pre-SMA cluster (red dots) and regression line (black dotted line). PreCG = precentral Gyrus; pre-SMA = pre-supplementary Motor Area.

***Data Quality Control***

*Paced tapping fMRI task*

Behavioural responses were visually inspected, in addition to being automatically processed by custom MATLAB scrips. Rest blocks that contained more than 3 responses after the first 5 pacing tones were excluded from the fMRI data analysis; 2.1% of all rest blocks were excluded from the fMRI analysis. Tapping blocks that contained no responses or less than 80% of the expected responses (less than 28 taps out of 35) were also excluded from the fMRI analysis; 2.2% of all tapping blocks were excluded from the fMRI analysis. One participant was excluded from both the behavioural and fMRI analyses, because of inability to tap following the pacing tone. Due to motor impairment, the number of responses made during most of the tapping blocks were less than 80% of the expected number of responses and therefore excluded from the analysis.

Data from another participant were also excluded from the analysis of the behavioural performance and the regressions with performance, because of technical problems with the button box that affected the pre-training sessions only. In a small number of runs (8 out of 120 runs in total) behavioural responses were not recorded properly because of technical issues with the response box, although the participant seemed to have performed the task correctly after inspection of the fMRI activation patterns. The first level contrasts for tapping vs rest were inspected to ensure that the pattern of brain activity was similar to runs for which the responses were recorded correctly.

*Neurofeedback training*

The movements recorded using the pneumatic tubes attached to the upper and lower limbs were visually inspected for the presence of overt motion during the upregulation and baseline blocks. If overt motion was identified, then the blocks were excluded from the analysis; 3% of upregulation blocks and 3.8% of rest blocks were excluded for this reason.

***References***

1. Stout, J.C. et al. *Mov. Disord.* **29**, 1281–1288 (2014).

2. Tabrizi, S.J. et al. *Lancet Neurol.* **10**, 31–42 (2011).

3. Draganski, B. et al. *Nature* **427**, 311–312 (2004).

4. Zatorre, R.J., Fields, R.D. & Johansen-Berg, H. *Nat. Neurosci.* **15**, 528–536 (2012).

5. Tabrizi, S.J. et al. *Lancet Neurol.* **8**, 791–801 (2009).

6. Tabrizi, S.J. et al. *Lancet Neurol.* **11**, 42–53 (2012).

7. Tabrizi, S.J. et al. *Lancet Neurol.* **12**, 637–649 (2013).

8. Subramanian, L. et al. *J. Neurosci.* **31**, 16309–16317 (2011).

9. Weiskopf, N. et al. *J. Physiol.-Paris* **98**, 357–373 (2004).

10. Linden, D.E.J. et al. *PLoS ONE* **7**, e38115 (2012).

11. Scharnowski, F., Hutton, C., Josephs, O., Weiskopf, N. & Rees, G. *J. Neurosci.* **32**, 17830–17841 (2012).

12. Lutti, A., Thomas, D.L., Hutton, C. & Weiskopf, N. *Magn. Reson. Med.* **69**, 1657–1664 (2013).

13. Weiskopf, N. et al. *Brain Imaging Methods* **7**, 95 (2013).

14. Draganski, B. et al. *NeuroImage* **55**, 1423–1434 (2011).

15. Callaghan, M.F. et al. *Neurobiol. Aging* **35**, 1862–1872 (2014).

16. Helms, G. & Dechent, P. *J. Magn. Reson. Imaging* **29**, 198–204 (2009).

17. Lutti, A., Hutton, C., Finsterbusch, J., Helms, G. & Weiskopf, N. *Magn. Reson. Med.* **64**, 229–238 (2010).

18. Ashburner, J. & Ridgway, G.R. *Brain Imaging Methods* **6**, 197 (2013).
